# Pure global amusia in a professional opera singer

**DOI:** 10.1007/s10072-026-08976-8

**Published:** 2026-04-11

**Authors:** Konstantinos Priftis, Bruno L. Giordano

**Affiliations:** 1 Department of General Psychology, University of Padua, Via Venezia, 8, 35131 Padua, Italy; 2 Institut de Neurosciences de La Timone (INT), UMR 7289, CNRS and Aix Marseille Université, Marseille, France

Amusia, the inability to process music, typically results from lesions to the right cerebral hemisphere, involving a dedicated network that includes the temporal, parietal and frontal lobes [1]. Amusia is typically striking, but it becomes even more remarkable when professional musicians are affected (e.g., [2]). Here, we reported on MK, a professional opera singer who after a right-hemisphere stroke lost her ability to sing in tune and to identify familiar musical pieces, despite her former acclaimed career.

At the time of testing, MK was a 41-year-old, right-handed female. At the age of five, she was affected by meningitis, which was successfully treated. Later, she was diagnosed with cardiopathy. MK had 13 years of formal education (upper-secondary diploma: music conservatory). She successfully graduated from the conservatory and started a brilliant career as a professional opera singer.

At the age of 40, MK was admitted to the hospital after the acute onset of a left hemi-syndrome because of a hemorrhagic stroke. A CT scan revealed an isolated right parietal hyperdense lesion signaling the presence of a hematoma, after an aneurysm rupture, with no other lesions identified. In the following months after the onset of her stroke, MK received motor rehabilitation for the treatment of her left-sided hemiparesis. Her spasticity was treated with Dantrolene sodium. Her mood was depressed and she reported sleeping difficulties. Therefore, MK was treated with Paroxetine and Triazolam.

We tested MK, 8.5 months after the onset of her lesion; she gave her informed consent to participate to the study. She was alert and perfectly oriented to space, time, and personal information. For instance, she could autonomously move among the sectors of the hospital, and she accurately recalled important information regarding her family. Language comprehension was perfect. Language production was fluent and syntactically correct. MK never produced phonetic, phonemic, morphological, verbal, or semantic paraphasias. Her speech was not aprosodic, but it occasionally appeared emotionally flat. Both her reading and writing abilities were intact. MK had an intact visual field and showed no sign of visual extinction upon simultaneous double stimulation. Finally, she never showed signs of hearing deficits.

Following her stroke, MK realized that she could no longer identify melodies and sing. She stated that this was astonishing and hard to believe. The fact that MK was fully aware of her deficit excluded the presence of psychodynamic factors underlying her behavior. When asked to sing, MK was largely out of tune. These signs were strongly indicative of amusia [1]. To further investigate her singing repertoire, we asked MK to sing some intervals (e.g., second, third, fourth, etc.) and music scales (i.e., chromatic, minor, major, and jazz). Finally, we asked MK to sing parts of three operas, from her previous repertoire (i.e., *“Mon coeur s'ouvre à ta voix”* by Camille Saint-Saëns, *“Habanera”* by Georges Bizet, and *“Stride la vampa”* by Giuseppe Verdi). On all these tasks, her singing remained highly inaccurate and out of tune. MK was perfectly aware of her difficulties resulting in a depressed mood.

We asked a control professional opera singer to evaluate recordings of MK’s performance^1^ on a seven-point Likert scale (1 = worst performance: no music skills; 7 = best performance: perfect music skills). Her performance was so impaired that, after hearing MK singing, the control singer expressed disbelief that MK had formerly been a professional opera singer. Coherently, his average evaluation scores for the singing performance of MK were: 4/7 for music intervals, 3/7 for scales, and 2.3/7 for the three operas. Importantly, the low scores for MK’s singing performance were due to melodic issues, because she was perfectly able to read music scores aloud, while keeping the correct rhythm and meter.

MK’s performed normally on tasks for assessing naming of environmental sounds, pitch perception, loudness perception, tempo perception, sound localization, rhythm perception, timbre perception, musical instruments naming, identification of ascending and descending scales (major vs. minor: see Supplemental data 1: https://osf.io/mz5nh/files). By contrast, her performance was impaired on tasks for testing music intervals perception, tone duration perception, and chord identification (i.e., distinguishing major and minor chords; see Supplemental data 1: https://osf.io/mz5nh/files).

To test her ability to recognize and identify famous operas, we presented MK with the following ones: *“La Traviata”* by Giuseppe Verdi, *“La Bohème”* by Giacomo Puccini, *“Carmen”* by Georges Bizet, *“Don Giovanni”* by Wolfgang Amadeus Mozart, *“Rigoletto”* by Giuseppe Verdi, and *“Tosca”* by Giacomo Puccini. All operas were presented without lyrics, to avoid recognition based on linguistic information. MK was able to immediately recognize all the operas as familiar ones (e.g., *“I know this! I have sung it before!”*). By contrast, she was unable to identify and name any of them. Nonetheless, MK was able to describe and name all these operas by associating them to their authors, through semantic cues (e.g., *“Please tell me what are Verdi’s or Puccini’s main operas”*).

In one instance, we re-presented MK with one opera (i.e., “Tosca”), but this time with the lyrics. MK was able to identify the opera through the lyrics. Furthermore, MK showed signs of implicit (i.e., unconscious) processing. Indeed, when she found herself unable to identify a musical piece (i.e., Beethoven’s ninth symphony), she stated: *“I do not know what this piece is, but it reminds me of Kubrick's film “A Clockwork Orange”*; the ninth symphony, indeed, was a part of the film’s soundtrack.

To sum up, MK showed signs of severe amusia. She effortlessly recognized all the operas, but she failed to identify them. We could hypothesize that her main input deficit was due to a disconnection between an intact store for known operas (i.e., a melody input store subserving familiarity) and an intact general-purpose, semantic memory (i.e., she was able to verbally produce semantic information about the same operas and to name them). A more peripheral input deficit can be excluded, because MK was not deaf. Furthermore, her performance was intact in perceiving speech, environmental sounds, and most aspects of peripheral melody processing.

We can hypothesize that her main output deficit could not be attributed to a semantic memory deficit, because MK could have access to all the requested operas (e.g., she produced correctly the lyrics) on verbal command of the examiner. Therefore, MK difficulties in singing might reside at a post-semantic level (e.g., a melody output store or its connection with more peripheral motor mechanisms). Finally MK’s output deficit could not be attributed to peripheral oral motor dysfunction, because she never showed signs of oral incoordination or paresis. This putative model, for melody processing, can be seen in Fig. 1 (for similar models, see [3], for praxis processing; see [4], for language processing).

**Fig. 1:**
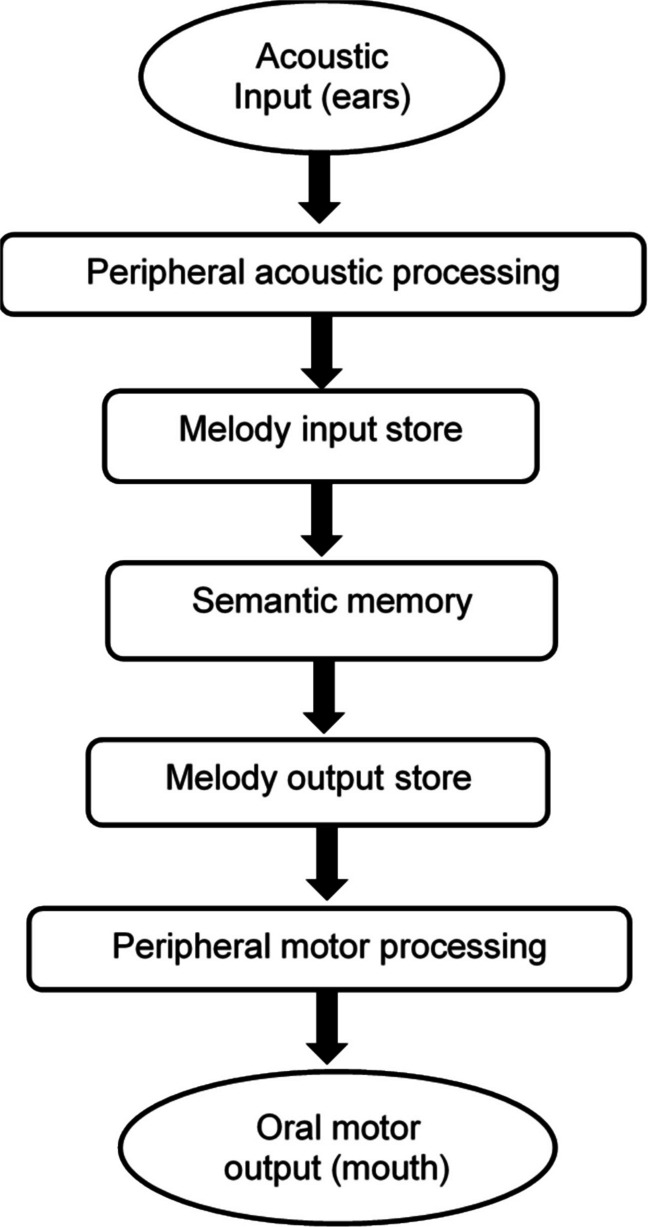
A model for melody processing. *Peripheral acoustic processing:* a general-purpose, peripheral module for processing all types of acoustic input (e.g., music, environmental sounds, speech, etc.). *Melody input store:* a module where all known/recognizable melodies are stored. *Semantic memory:* a general-purpose semantic store containing encyclopedic knowledge and concepts. *Melody output store:* a module where all known/producible melodies are stored. *Oral motor output:* a general-purpose peripheral module underlying oral output (e.g., singing, speaking, etc.)

Despite her severe amusia, MK performed normally on a variety of neuropsychological tests for assessing general cognitive status, spatial attention, short-term memory, long-term memory, and executive functions including verbal/non-verbal reasoning (Table 1). Moreover, MK’s performance was errorless on an ad hoc made battery for assessing number processing (i.e., number comparison, parity judgment, reading-writing-repeating spoken and written number words/Arabic digits), and calculation (i.e., comprehension of operation signs, arithmetic facts, approximate and exact calculation). To sum up, MK did not show any sign of topographical disorientation, neglect (personal, peripersonal, extrapersonal, imaginal), agnosia, constructional apraxia, short-term and working memory deficits,^2^ amnesia, acalculia, aphasia, alexia, agraphia, oral apraxia, or dysexecutive syndromes. By contrast, MK clearly showed deficits consistent with severe amusia.

**Table 1:** MK’s neuropsychological profile for non-musical cognitive functions

Cognitive domain/Test	Reference	Raw score	Adjusted score	Equivalent score	Cut-off
Global mental state (Mini-mental State Examination)	Folstein et al. [5]	29/30			< 24/30
Personal neglect	Informal testing	Absent. The patient was perfectly able to explore both sides of her body			
Peripersonal neglect	Wilson et al. [6]	134/146			< 129/146
Extrapersonal neglect	Informal testing	Absent. The patient was perfectly able to describe both sides of the examination room			
Imaginal neglect (drawing from memory)	Wilson et al. [6] and additional drawings	Absent. All her drawings from memory were symmetrical			
Constructional apraxia (copy drawing)	Spinnler and Tognoni [7]	12	10.5	2/4	0/4
Oral apraxia	Informal testing	The patient was perfectly able to produce, on command, non-linguistic oral movements (blowing, kissing, whistling, etc.)			
Auditory-verbal short-term memory (digit span forward)	Monaco et al [8]	6	5.41	3/4	0/4
Auditory-verbal working memory (digit span backward)	Monaco et al. [8]	3	2.58	0/4	0/4
Semantic verbal fluency	Novelli et al. [9]	43	39	4/4	0/4
Phonemic verbal fluency	Novelli et al. [9]	35	30.5	3/4	0/4
Rey 15 words: immediate recall	Carlesimo et al. [10]	54	47.8	4/4	0/4
Rey 15 words: delayed recall	Carlesimo et al. [10]	11	9.1	4/4	0/4
Verbal reasoning	Spinnler and Tognoni [7]	51	44	2/4	0/4
Non-verbal reasoning (Raven Colored Matrices)	Carlesimo et al. [10]	32	28.8	4/4	0/4

To the best of our knowledge, this is the first report of a professional opera singer who became amusic (but see [2], for an amusic tango singer). Her total inability to identify famous operas and her deficits in singing were in clear contrast with her normal performance on a variety of neuropsychological tests for assessing non-musical cognitive functions. Because MK showed both receptive deficits (i.e., inability to identify famous operas) and expressive deficits (inability to sing correctly the operas), we propose the term “global amusia”. This is by analogy to global aphasia (i.e., co-presence of receptive and expressive deficits in language processing). Note that Wilson et al. [11] were the first -and only- to use the term global amusia, but without defining it. Because MK’s amusia was pure (i.e., no other cognitive deficits were present), we termed her deficit as “pure global amusia”.

We conclude that the occurrence of amusia in professional musicians provides a unique window into the neuropsychological underpinnings of expert music processing and performance. Consistently with the findings from some previous cases, our results suggest the presence of a highly modular network for music processing [12]: [2], supported by the right cerebral hemisphere [1]. Because amusia can be the cause of severe emotional problems, in experienced musicians who cannot perform professionally anymore, there is an increased demand for rehabilitation in these patients.

## Data Availability

All stimuli, data, and results are available on Open Science Framework: https://osf.io/mz5nh/files

## Footnotes

1 MK’s singing output is available, from the corresponding author, upon motivated request of qualified scholars and clinicians.

2 The only exception was her abnormal performance on the digit span backward test Table 1. Nevertheless, MK was errorless on a much more difficult task of working memory (i.e., serial backward counting by seven, [5]).
